# Luminescence Lifetime-Based Water Conductivity Sensing Using a Cationic Dextran-Supported Ru(II) Polypyridyl Complex

**DOI:** 10.3390/s25010121

**Published:** 2024-12-28

**Authors:** Ya Jie Knöbl, Lauren M. Johnston, José Quílez-Alburquerque, Guillermo Orellana

**Affiliations:** Chemical Optosensors & Applied Photochemistry Group (GSOLFA), Department of Organic Chemistry, Faculty of Chemistry, Complutense University of Madrid, 28040 Madrid, Spain; yaknoebl@ucm.es (Y.J.K.); ljohns01@ucm.es (L.M.J.); joquilez@ucm.es (J.Q.-A.)

**Keywords:** electrical conductivity, water, luminescent sensors, luminescence lifetime, environmental monitoring

## Abstract

Water conductivity sensing relies universally on electrical measurements, which are subject to corrosion of the electrodes and subsequent signal drift in prolonged in situ uses. Furthermore, they cannot provide contactless sensing or remote readout. To this end, a novel device for water conductivity monitoring has been developed by employing a microenvironment-sensitive ruthenium complex, [Ru(2,2′-bipyridine-4,4′-disulfonato)_3_]^4−^, embedded into a quaternary ammonium functionalized cross-linked polymer support. The degree of swelling of the latter, which leads to a change in the emission lifetime, depends on the water conductivity. The sensor displays a reversible response (2 min ≤ *t*_90_ ≤ 3 min) and has been shown to be stable for >65 h of continuous monitoring of 0.8–12.8 mS cm^−1^ KCl solutions. Changes to the cation do not affect the sensor response, while changes to the anion type induce small effects. Variations in the dissolved O_2_ or temperature require corrections of the response. The sensor can be interrogated alongside dissolved O_2_ and pH luminescent sensors based on the same family of indicator dyes to exploit the definite advantages of luminescence lifetime-based detection.

## 1. Introduction

Measurement of the electrical conductivity (κ) of water is of high importance both for environmental monitoring and industrial process control. The electrical conductivity of water is directly linked to the amount of dissolved ions, a pollutant of groundwater leading to the salinization of drinking water, rivers, and other natural water bodies [1,2,3,4]. This parameter is also useful to assess the total dissolved solids (TDS) present in natural waters, to determine the degree of mixing between fresh- and seawater, to gauge the relative contribution of precipitation and subsurface water to a water body, or to estimate the dilution coming from a stream discharge, to name a few goals [5].

Defined as the reciprocal of resistivity, the electrical conductivity of the sample is measured by using a two- or four-electrode sensor and amperometric or potentiometric measurements, respectively [6]. Since the electrical conductivity of aqueous solutions depends intrinsically on the nature of the dissolved ions, only a compound measurement is possible [7]. While displaying definite advantages such as cost, straightforward implementation, low maintenance, ease of miniaturization, and wide measurement range, the electrodes are not infallible. Salt deposits and corrosion lead to signal drift and may underestimate the amount of dissolved ions at high concentrations [6,8,9]. Alternative specialized methods to measure conductivity include optical techniques such as refraction, fiber grating, surface plasmon resonance, or interferometry [10,11,12,13,14,15,16,17,18,19]. These methods usually focus on ionic strength or salinity determinations since the first parameter is more relevant in biological systems while the second one is only relevant for seawater. Table S1 in the Supporting Information summarizes the most relevant optical sensors for ionic strength or salinity described in the literature and compares their performance to that of the novel sensor reported in this work.

Despite the advantages of some optical sensors for ionic strength or salinity, such as contactless readout and resiliency against corrosion, all those reported so far are designed as unique sensors with their own particular detection system. Therefore, it is difficult to combine them with another optical sensor for a water quality parameter that uses the same detector and operates under the same principle. This combination would result in bulkier multiparametric sensor arrays that house the different optoelectronics. To facilitate integration, it is worth designing optical sensors for different analytes based on the very same operating principle.

Luminescence lifetime-based sensors for dissolved O_2_, another important water quality parameter, have phased out the classical electrochemical Clark electrode [20]. Capitalizing on the robustness of measuring this intensive property of a membrane-embedded indicator dye as a function of the waterborne O_2_, the sensor is not susceptible to interferences induced by other dissolved gases, sensor contamination, indicator photobleaching or leaching, or detector or excitation light source aging. Outside the lab, luminescence lifetime can be measured either in the time domain with the so-called rapid lifetime determination technique or, more commonly, in the frequency domain through phase shift *ϕ* determinations [20]. The latter is related to the emission lifetime by Equation (1),

(1) $$\tan\phi =2\pi f\tau_{\phi }$$

where *f* is the modulation frequency of the excitation light and *τ_ϕ_* is the corresponding emission lifetime to the measured *ϕ*. Using well-established optoelectronic systems for dissolved O_2_ sensing based on phase shift monitoring, this work introduces a novel conductivity sensor.

A ruthenium(II) polypyridyl complex has been selected as the luminescent dye to develop the new sensor due to the unique features of this class of indicator molecule, namely, large Stokes shift with absorption in the blue and emission in the red regions, sub-µs emission lifetimes, structural versatility toward their immobilization on solid supports, and their tuneability to the analyte, as well as photochemical and thermal stability [21,22]. However, as our goal was to design a non-specific indicator dye for ionic species in water, we decided to take advantage of their environment-sensitive luminescence [23] and embed the probe into a polyelectrolyte. The extent of the electrostatic repulsion between the charged polymer chains of the latter is determined by the concentration and nature of the dissolved ions [24,25,26,27]. This change in the polymer swelling leads to a local polarity variation that can be probed by the bound indicator dye. Actually, ruthenium(II) polypyridyl complexes have already been used to probe the microenvironment of polyelectrolytes [28,29,30,31,32]. This photophysical working principle differs from the photochemical quenching of ruthenium(II) polypyridyls by O_2_ (energy transfer through the Dexter mechanism) underlying the luminescent sensors for dissolved O_2_ [20,33,34].

## 2. Materials and Methods

Reagents, solvents, and utilized instruments are described in the Supporting Information.

### 2.1. Synthesis of Tetrasodium Tris(2,2′-bipyridine-4,4′-disulfonato)ruthenate(II) (Na_4_[Ru(bpds)_3_])

The precursor disodium 2,2′-bipyridine-4,4′-disulfonate (bpds) ligand is prepared from 2,2′-bipyridine via 2,2′-bipyridine-4,4′-disulfonic acid *N,N*′-dioxide according to the literature [35,36] with the following slight modification. For the reduction of the *N*-oxidized ligand, 5.63 g of the disodium salt of 2,2′-bipyridine-4,4′-disulfonic acid *N,N*′-dioxide (14.36 mmol), and the Pd/C catalyst (650 mg, 10 mol%) are placed into a two-necked flask and argon is flushed before 55 mL of anhydrous methanol is added dropwise to the mixture. After 15 min of stirring at room temperature, anhydrous ammonium formate (9.6 g, 152.4 mmol) is added and the reaction mixture stirred for another 15 min before heating to 40 °C for 6 h. The reaction mixture is allowed to cool to room temperature before being filtered and the solid washed with methanol. The resulting filtrate is rotavaporated until dryness, redissolved in a small amount of methanol-H_2_O (4:1 *v*/*v*) mixture, and the product precipitated upon addition of absolute ethanol. The final white solid is isolated by filtration and vacuum-dried (72% yield).

The tetrasodium salt of tris(2,2′-bipyridine-4,4′-disulfonato)ruthenate(II) (Na_4_[Ru(bpds)_3_]) complex is prepared using a modification of a standard route to homoleptic Ru(II) complexes. RuCl_3_ hydrate (25 mg, 0.12 mmol) and bpds (135 mg, 0.37 mmol) are dissolved in 5 mL of abs. EtOH-H_2_O-glycerin (3:1:1 *v*/*v*) mixture, placed in a 10 mL microwave reaction vessel and purged with argon. The mixture is then microwaved at 150 °C for 1.5 h and the resulting reaction mixture rotavaporated until only the glycerin remains. Then, 25 mL of EtOH is added to precipitate the complex and the solution is stored at 4 °C overnight. The precipitate is filtered out and dried under reduced pressure. To remove the glycerin, the complex is redissolved in water and reprecipitated with EtOH, filtered, and vacuum-dried (0.1 Torr) to obtain the final product in 87% yield. ^1^H NMR (300 MHz, DMSO-*d*_6_) δ (ppm): 8.53 (d, *J* = 1.5 Hz, 6 × H3), 7.79 (d, *J* = 5.8 Hz, 6 × H6), 7.59 (dd, *J* = 5.8, 1.7 Hz, 6 × H5). ESI(–) *m*/*z*: calculated for RuC_30_H_18_N_6_O_18_S_6_Na_2_^2−^: 544.9, found: 545.1; calculated for RuC_30_H_18_N_6_O_18_S_6_Na^3−^: 355.6, found: 355.8.

The tetrabutylammonium (TBA) salt of [Ru(bpds)_3_] is prepared by weighing 25 mg of (Na_4_[Ru(bpds)_3_] (0.022 mmol) and 25 mg (0.090 mmol) of tetrabutylammonium chloride (TBA-Cl) and dissolving them in water. The solution was then extracted with dichloromethane and the organic solvent was rotavaporated. ^1^H NMR (300 MHz, acetone-*d*_6_) δ (ppm): 8.73 (d, *J* = 1.2 Hz, 6 × H3), 8.13 (d, *J* = 5.7 Hz, 6 × H6), 7.71 (dd, *J* = 5.7, 1.6 Hz, 6 × H5), 3.45–3.41 (m, 24H), 1.78 (dt, *J* = 15.9, 7.8 Hz, 49H), 1.39 (h, *J* = 7.4 Hz, 52H), 0.94 (t, *J* = 7.3 Hz, 74H).

### 2.2. Fabrication of the Sensitive Terminal

To incorporate the indicator dye into the Sephadex^®^ QAE A-50 resin beads (40 to 120 µm diameter; Cytiva, Marlborough, MA, USA), 1 mL of a 1.0 mg mL^−1^ aqueous solution of Na_4_[Ru(bpds)_3_] is diluted with 1 mL of a 100 mmol L^−1^ KCl aqueous solution (=12.8 mS cm^−1^) and then added dropwise to 25 mg of dry resin (5% loading; the corresponding amounts have been used for other loading levels). The resin is heated at 80 °C for 1.5 h on a heating plate for outgassing and, after cooling to room temperature, the dyed resin is stored in the dark.

To manufacture the sensor spot, two 9 mm dia. discs of 200 µm thick Mylar^®^ (Qbix, The Hague, The Netherlands) are cut, one with a precut 5 mm dia. center hole. The two discs are glued together with a transparent anti-mold modified silicone adhesive (MS Professional Polymer Sealant Adhesive, Fischer, Waldachtal, Germany) and dried overnight. The next day, ca. 1 mg of the dyed QAE resin is placed in the shallow well created by the center hole. A protective membrane of hydrophilic poly(tetrafluoroethylene) (h-PTFE, H100A013A, Advantec Toyo Kaisha, Tokyo, Japan; 1 µm pore size, 35 µm, 73 mL min^−1^ cm^−2^ max. flow rate) is placed on top of the disc and glued with the MS adhesive only to the underside of the disc. The assembled sensitive terminal is allowed to dry at room temperature for 4 h before being stored in 0.1 mol L^−1^ KCl solution for at least 24 h before any measurement.

## 3. Results and Discussion

### 3.1. Selection of the Complex and Solid Support

Upon consideration of the requirements of the ion-sensitive polymer support outlined in Section 1, Cytiva’s Sephadex^®^ QAE A-50 resin containing quaternary aminoethyl functional groups was chosen. These polymer beads are commercially available, compatible with autoclaving, structurally stable, and remain transparent after hydration. The diethyl(2-hydroxypropyl)aminoethyl groups on the resin chains provide a positive charge over the entire pH range, causing the resin to swell or shrink depending on the ionic strength of the water (Figure S1, Supporting Information) [37]. In order to firmly bind the Ru(II) indicator dye to the resin, the former must contain as many negative charges as possible, and these charges must also be independent of pH. Therefore, the tris(2,2′-bipyridine-4,4′-disulfonato)ruthenate(II) (Na_4_[Ru(bpds)_3_]) complex was prepared. The alternative and well-known highly luminescent homoleptic 1,10-phenantroline-4,7-diphenyldisulfonate Ru(II) complex was not selected in order to reduce the effect of dissolved O_2_ on the sensor luminescence due to its more than seven-fold longer excited state lifetime [36]. Because of the significant electron-withdrawing nature of the sulfonate groups [38], the synthesis of the complex from the non-commercial ligand had to be assisted by microwaves, since simple heating takes more than 40 h until completion.

### 3.2. Photophysical Properties of the Indicator Dye

The absorption and emission spectra of [Ru(bpds)_3_]^4−^ in solvents of different polarities were measured to determine their sensitivity to the microenvironment. To dissolve the highly polar complex in non-aqueous solvents, tetrabutylammonium (TBA) was exchanged for sodium counter-cations. Then, we used the Lippert–Mataga (L-M) model [39] to investigate the potential relationship between the Stokes shift (*ν*_abs_-*ν*_em_) of the indicator dye and the solvent polarity as expressed by the orientation polarizability (Δ*f*). This model assumes that the solvent is a continuous medium in which the solute is in a spherical cavity without any mutual specific interactions. According to the L-M model, the relationship should follow Equation (2),

(2) $$\nu_{abs}-\nu_{em}=\frac{2}{hc}\left(\frac{\varepsilon -1}{2\varepsilon +1}+\frac{n^{2}-1}{2n^{2}+1}\right)\frac{(\mu_{e}-\mu_{g}{)}^{2}}{a^{3}}+const.=\frac{2}{hc}∆f\frac{(\mu_{e}-\mu_{g}{)}^{2}}{a^{3}}+const.$$

where *ε* is the solvent dielectric constant, *h* the Planck constant, *c* the speed of light, *a* is the radius of the solvent “cavity” where the solute molecule dwells, *µ_e_* and *µ_g_* the dipole moments of the luminophore in the excited and ground states, respectively, and *n* the solvent refractive index.

As shown in Table 1, the Stokes shift increases with Δ*f*, showing the ability of [Ru(bpds)_3_]^4−^ to probe the polarity of its microenvironment. In fact, the sensitivity of this luminescent probe (L-M slope = 5926 cm^−1^) is larger than that of related indicator dyes such as [Ru(bpy)_3_]^2+^ (L-M slope = 3670 cm^−1^; bpy = 2,2′-bipyridine) [40] or [Ru(4,4′-dinonyl-bpy)_3_]^2+^ (L-M slope = 2061 cm^−1^) [41]. The electron-withdrawing character of the sulfonate groups would be responsible for the enhanced sensitivity due to the larger dipole moment of [Ru(bpds)_3_]^4−^ in the emissive ^3^MLCT excited state (all three Ru(II) complexes have a zero dipole moment in their ground state due to their symmetry).

**Figure 1:**
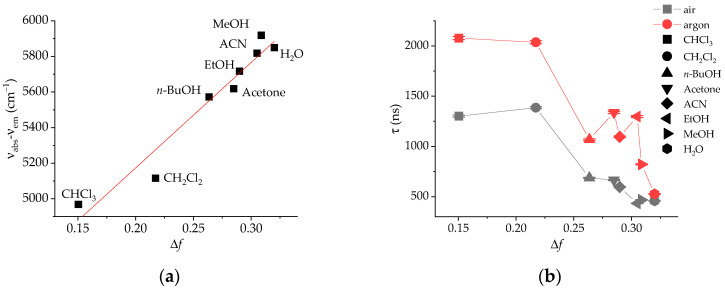

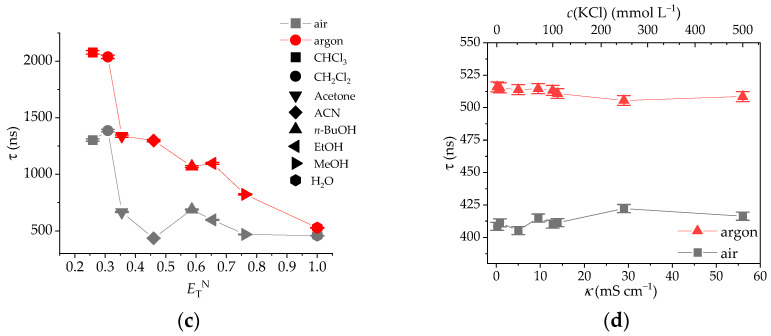
(**a**) Lippert–Mataga plot of [Ru(bpds)_3_]^4−^ in different solvents. The red line represents the linear fit. (**b**,**c**) Emission lifetime of (TBA)_4_[Ru(bpds)_3_] under air and under argon at room temperature in selected solvents, as a function of the L-M parameter (**b**) or the normalized *E*_T_^N^ parameter (**c**). (**d**) Luminescence lifetime of [Ru(bpds)_3_]^4−^ in solutions of different KCl concentrations under air and under argon at (23 ± 1) °C. The conductivity (*κ*) values for 250 mmol L^−1^ and 500 mmol L^−1^ were taken from ref. [45].

After the steady-state measurements, the luminescent lifetime (τ) of the complex was also measured in aerated and O_2_-free conditions to evaluate the environmental sensitivity of this parameter, which will be used for lifetime-based sensing (Figure 1a,b). In addition to the L-M model, the (normalized) Reichardt solvent polarity parameter, *E*_T_^N^, was also tested with a similar trend [39]. In the absence of O_2_ quenching and regardless of the polarity scale, the luminescence lifetime of [Ru(bpds)_3_]^4−^ dramatically decreases with the solvent polarity, as shown for other 4,4′-disubstituted bipyridine Ru(II) complexes [23]. Taking into account the important electron-withdrawing features of the -SO_3−_ groups, the excited state lifetime of the corresponding homoleptic Ru(II)-X_2_-bpy complexes will be controlled by the ^3^MLCT-^3^MC energy gap [42,43,44]. The non-luminescent shorter-lived ^3^MC state is populated by thermally activated crossing from the ^3^MLCT at room temperature, and the contribution of this deactivation pathway depends on the actual separation between the metal *e*_g_ and the ligand π* orbitals. This gap is smaller with electron-deficient ligands, and a strong hydrogen bonding to the solvent molecules will certainly increase the electron deficiency of the bpy-disulfonate ligands. The situation is more complicated in the presence of O_2_ due to the different solubility and quenching constants of this gas in the various solvents.

We also investigated the effect of salt (KCl) on the photophysical features of [Ru(bpds)_3_]^4−^ in water. Figure S2 in the Supporting Information depicts the absorption and emission spectra of the complex in solutions of different KCl concentrations. No significant difference was found in the investigated range because the wavelength of the emission maximum only shifts ca. 1.5 nm when the concentration changes from 1 to 500 mmol L^−1^. The emission lifetime of [Ru(bpds)_3_]^4−^ was also measured in both aerated and deoxygenated solution (Figure 1d). In the absence of O_2_, τ shows a slight but significant decrease upon increasing KCl concentration in agreement with the more polar environment in the presence of a high concentration of salt. The opposite behavior occurs in the oxygenated solutions due to the lower O_2_ concentration [46] and therefore weaker quenching in increasingly saline solutions, which obscures the polarity effect.

### 3.3. Photophysical Features of the Polymer-Supported Indicator Dye

As we discussed above, the highly charged [Ru(bpds)_3_]^4−^ complex is efficiently and quickly immobilized into the cationic resin beads (Figure S4). To that end, an aqueous solution of the indicator dye is added to the dry beads, and the suspension is heated for outgassing the trapped air and to allow for homogenous wetting. In fact, the indicator dye must be dissolved in a KCl solution to avoid the fracture of the polymer network that occurs in pure water due to excessive swelling. In an attempt to increase the hydrophilicity of the resin and therefore to increase the chloride exchange rate, the beads were autoclaved for 30 min at 120 °C before the addition of [Ru(bpds)_3_]^4−^. However, as no noticeable difference in the sensitivity of the polymer-supported dye was found, this additional treatment was left out.

**Figure 2:**
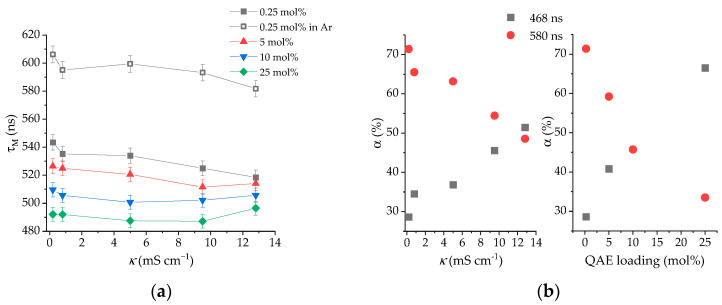
(**a**) Pre-exponentially-weighted emission lifetime (τ_M_) of the dyed QAE resin at different loading levels under aerated conditions, unless otherwise stated. τ_M_ was calculated according to the equation τ_M_ = ΣB_i_τ_i_/ΣB_i_ where B_i_ and τ_I_ are the parameters of the biexponential best fit of the luminescence decay to the kinetic equation: *I*_L_(*t*) = A_0_ + ΣB_i_exp(−*t*/τ_i_). (**b**) Relative contribution of the two lifetime components at different conductivity levels in the 0.25 mol% dyed QAE resin (left) and relative contribution of the two lifetime components at different dye loading levels of the QAE resin at 0.2 mS cm^−1^ (right).

The suspended beads at 0.25 mol% dye loading were exposed to KCl solutions of increasing concentration in the 0.2–12.8 mS cm^−1^ range, and their luminescence lifetime was measured under aerated and deoxygenated conditions (Figure 2a). The emission lifetime of the immobilized [Ru(bpds)_3_]^4−^ is always longer than that of the free complex in solution due to the restricted access of O_2_ to the excited polymer-bound dye. This restriction is evidenced by the smaller O_2_ quenching constant of the latter (Figure S6, Supporting Information) [20,47]. The probe shows a small but significant decrease in its τ with increasing conductivity under both aerated and O_2_-free conditions (Figure 2a). This result confirms that indeed the probe is sensitive to changes in conductivity and not just to changes in the dissolved O_2_ level as a consequence of the salinity effect [46].

A closer look at the components of the excited state decay of the immobilized complex reveals two distinct lifetimes of 468 ns and 580 ns (Figure 2b). We propose that the longer lifetime corresponds to a more tightly bound [Ru(bpds)_3_]^4−^, i.e., a binding site with four quaternary ammonium groups, while the shorter lifetime suggests a complex “loosely” bound to a site with fewer ammonium groups. A biexponential global analysis of the excited state decay measurements at different conductivities shows that the contribution of the loosely bound complex increases with increasing salt concentration (Figure 2b). Upon the addition of chloride, [Ru(bpds)_3_]^4−^ is shifted from the tight binding sites to the loose binding sites. This displacement can also be observed when higher loading levels, namely, 5, 10, and 25 mol%, were tested to optimize the sensor response versus the amount of indicator dye (Figure 2a). At 0.2 mS cm^−1^, the distribution of the two binding sites between the different loading levels is only controlled by the amount of the immobilized complex (Figure 2b). However, at a 10 mol% loading level, more of the complex is located at loose binding sites than at tight binding sites, limiting the sensitivity of the probe. Therefore, to balance between low loads for better sensitivity and high loads for better s/n ratio, the 5 mol% indicator dye loading was chosen and used throughout subsequent testing.

### 3.4. Sensor Fabrication and Testing

As described in Section 2, the sensing terminal was assembled by placing a small amount of dyed wet resin beads onto the transparent Mylar well and covering them with a hydrophilic PTFE (h-PTFE) membrane (Figure S7, Supporting Information). Since the resin beads should be able to freely swell and shrink, care must be taken when gluing the membrane to the underside of the Mylar window to avoid any contact between the beads and the glue. The sensing terminal was conditioned by placing it into a 12.8 mS cm^−1^ KCl solution for at least 24 h before the start of the measurements.

**Figure 3:**
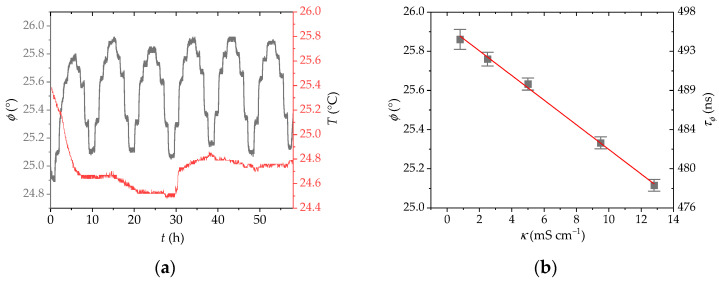

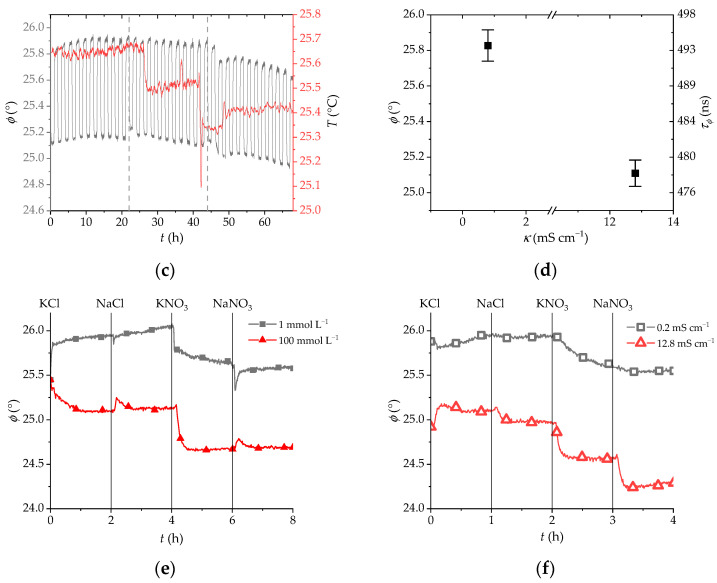
(**a**) Response of the conductivity sensor to different conductivity levels (0.8, 2.5, 5.0, 9.5, and 12.8 mS cm^−1^) set by 6, 17.5, 35, 70, and 100 mmol L^−1^ KCl solutions. (**b**) Calibration plot of the conductivity sensor from the data in (**a**); uncertainty bars are calculated for *n* = 6. The red line represents the linear least squares regression (*y* = 25.93–0.063*x*; *R*^2^ = 0.998). (**c**) Sensor response to conductivity cycling between 0.8 and 12.8 mS cm^−1^ set by 6 and 100 mmol L^−1^ KCl solutions; readings are not corrected for temperature changes. The grey dashed lines represent manual changes in the sample solutions, which may induce artificial signal variations not related to the sensor response. (**d**) Average sensor response from the data in (**c**) (*n* = 34). (**e**) Sensor response to different salts at a constant concentration of 1 mmol L^−1^ and 100 mmol L^−1^. (**f**) Sensor response to different salts at a constant conductivity of 0.2 mS cm^−1^ and 12.8 mS cm^−1^.

The sensor-reversible response to different KCl concentrations was tested (Figure 3a); it was shown to be sensitive to changes in the conductivity up to at least 12.8 mS cm^−1^ with a sensitivity of 0.063° phase shift/mS cm^−1^ (Figure 3b). Further decrease in the phase shift is observed above this conductivity, but at the expense of some leaching of the indicator dye from loosely bound positions. When the sensor is cycled between 0.8 and 12.8 mS cm^−1^ (Figure 3c,d), it remains stable for at least 65 h considering that the signal is not corrected for temperature variations (precision is ±0.09° at 0.8 mS cm^−1^ and ±0.07° at 12.8 mS cm^−1^ with the standard parameters of the optoelectronic unit). Ultimately, the sensor stability is determined by the slow indicator leaching at high conductivity levels. The sensor response time (*t*_90_) is highly dependent on the sample flow rate: at a flow rate of 4.1 mL min^−1^, *t*_90_ for a change from 0.2 to 12.8 mS cm^−1^ is 3 min and *t*_−90_ is < 2 min while at 1.7 mL min^−1^, *t*_90_ = 5.7 min and *t*_−90_ = 5.2 min (Figure S8, Supporting Information). Therefore, diffusion of ions into the polymer beads is the rate-limiting step considering the membrane water flow rate (see Section 2).

A comparison between KCl and other monovalent salts, namely, NaCl, KNO_3_, and NaNO_3_ (Figure 3e,f), was performed to evaluate the sensor responsiveness toward other ions and not provide a specific response to either potassium or chloride ions. For the sake of comparison, the tests were conducted both under constant molar concentration (1 and 100 mmol L^−1^) and under constant conductivity (0.2 and 12.8 mS cm^−1^). Our results show that at low ion concentrations, only a change to the anion affects the response of the sensor. Cl^−^ and NO_3_^−^ have different solvation features in aqueous media and display distinct interactions with charged macromolecules. According to the Hofmeister series, NO_3_^−^ is regarded as chaotropic [48], so that it causes shrinking of the ionomer beads upon entering the polymer network, pushing the [Ru(bpds)_3_]^4−^ dye molecules into the loosely bound sites, resulting in a lower phase shift. At high salt concentrations, the sensor responsiveness toward the different salt-type changes. While under constant molarity, only the anion type induces a change (Figure 3c), under constant conductivity, the sensor is influenced by changes in both the salt nature and concentration (Figure 3d). The same phenomenon is observed in the classical electrical conductivity measurements. These differences are ultimately due to the different salt concentrations required to achieve constant conductivity.

**Figure 4:**
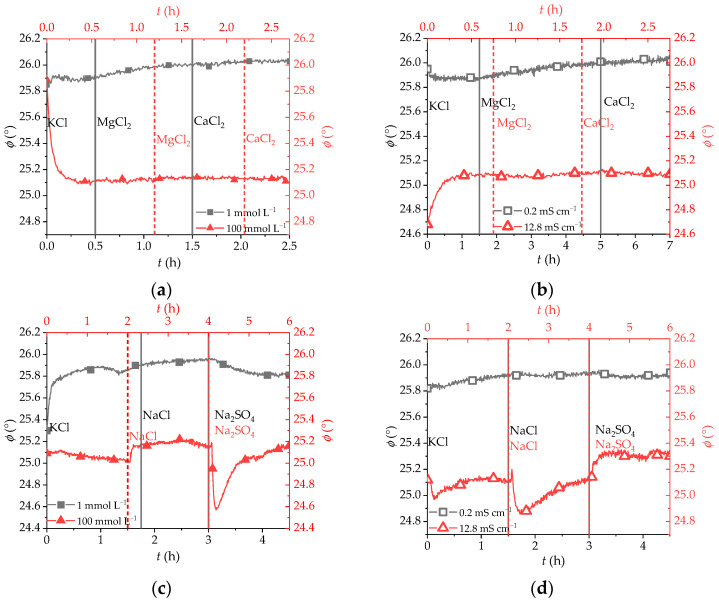
(**a**) Sensor response to different divalent cations at constant salt concentrations of 1 mmol L^−1^ and 100 mmol L^−1^. (**b**) Sensor response to different divalent cations at constant conductivities of 0.2 mS cm^−1^ and 12.8 mS cm^−1^. (**c**) Sensor response to SO_4_^2−^ at constant concentrations of 1 mmol L^−1^ and 100 mmol L^−1^. (**d**) Sensor response to SO_4_^2−^ at constant conductivities of 0.2 mS cm^−1^ and 12.8 mS cm^−1^. The vertical lines represent the moment when the concentration or the conductivity change was carried out (the black solid lines refer to the low salt concentration/conductivity while the red dotted lines correspond to the high salt concentration/conductivity).

To further investigate the sensor behavior, tests with MgCl_2_, CaCl_2_, and Na_2_SO_4_ divalent salts were conducted (Figure 4). Although the cations of the first two salts have a higher charge than K^+^, their effect on the sensor response is negligible both at low and high concentrations and regardless of their nature. This is not surprising since the positively charged polymer backbone repels the highly charged cations, and therefore the sensor output is unaffected. Furthermore, in the high ion concentration regimen, the lack of sensor sensitivity to the divalent salts is due to the inability of the resin to shrink further. As shown in Figure S1 (Supporting Information), the QAE Sephadex A-50 is only capable of shrinking until a chloride concentration of ca. 0.2 mol L^−1^ [37], an ionic strength value that the solutions of MgCl_2_, CaCl_2,_ and Na_2_SO_4_ exceed. The SO_4_^2−^ anion does not provoke any effect at low concentrations either because, as a kosmotropic ion [48], it does not penetrate the ionomeric network of the beads and cannot displace [Ru(bpds)_3_]^4−^ as efficiently as the chaotropic NO_3_^−^ or the Cl^−^ anions do, even though the SO_4_^2−^ is double-negatively charged.

**Figure 5:**
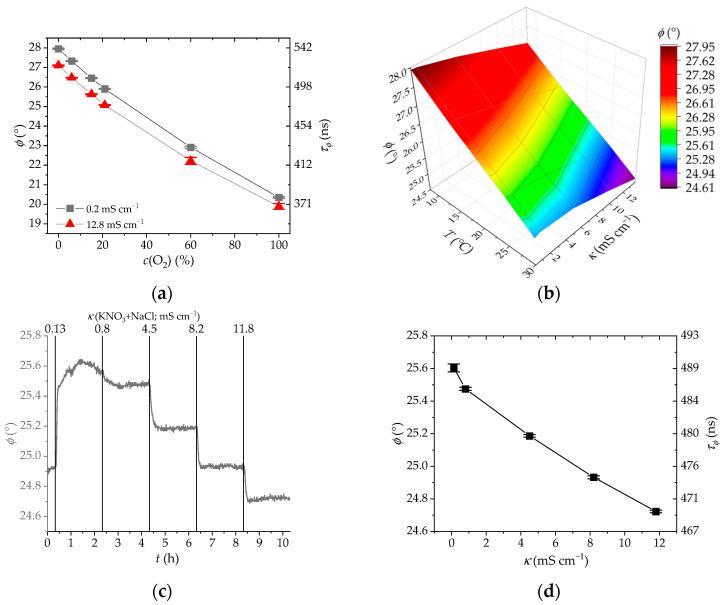
(**a**) Plot of conductivity sensor response to dissolved O_2_ from data in Figure S9a–f (Supporting Information) at 25 °C. (**b**) Temperature calibration of the conductivity sensor at 8, 15, 25, and 30 °C in the 0.2–13.9 mS cm^−1^ conductivity range. (**c**) Sensor response to a mixed solution of KNO_3_ and NaCl; the vertical lines represent the conductivity of the solutions. (**d**) Plot of conductivity sensor response to mixed salts from the data in (**c**).

We also tested the sensor response to other established water quality parameters such as dissolved O_2_ and temperature. We can observe in Figure 5a,b that the sensor shows some response toward the changes in either temperature or oxygen. These cross-sensitivities arise from the ruthenium complex dye, the luminescence of which is known to depend on these two parameters [21]. While the indicator dye in solution displays a moderate O_2_ effect (Figure 1d), the immobilized dye shows a 3-fold smaller response toward the O_2_ concentration changes (for instance, Δϕ is ~2° or 36 ns at 156 kHz in going from 0% to 21% O_2_ regardless of the salt concentration, see Figure S9a–f, Supporting Information and Equation (1)). The partial shielding from the O_2_ quenching provided by the polymer backbone is responsible for this difference, as discussed above.

The conductivity of aqueous solutions increases with increasing temperature due to higher ion mobility. At the same time, a temperature increase raises the rate constant of the thermally activated crossing to the non-radiative ^3^MC excited state, so that the measured phase shift becomes lower (Figure 5b) because of the shorter lifetime of the emissive excited state. Therefore, both dissolved O_2_ and temperature must be corrected when using the sensor in situ for water quality or industrial monitoring. Temperature can be corrected by adding or subtracting an offset from the measured phase shift, while the adjustment for dissolved O_2_ requires a correction function as has been described for chemical sensing with luminescent Ru(II) polypyridyls [49].

After testing the different pure salt solutions and cross sensitivities, one last study was performed by mixing different ions and investigating their combined effect on the sensor outcome. The sensor shows a similar response and sensitivity to the measurements obtained from pure KCl solutions (Figure 5c,d). The absolute phase shift is slightly lower due to the addition of the NO_3_^−^ anion; however, the sensitivity is comparable (1.5 mS cm^−1^ per 0.1° phase shift change, the same sensitivity as with the pure KCl solution).

## 4. Conclusions

Through the incorporation of a microenvironment-sensitive ruthenium complex into charged polymer beads, a new type of luminescence lifetime-based sensor for electrical conductivity measurements of water has been developed. Thanks to electrostatic immobilization, the negatively charged indicator dye is embedded into the polymer support to form the sensitive terminal. The sensor is capable of sensing conductivity changes regardless of the contributing ion, shows a sensitivity of 0.063°/mS cm^−1^, and is stable for at least 65 h under constant measurement and water flow. Despite its cross-sensitivity toward dissolved O_2_ and temperature, the sensor could be implemented in a multiparametric probe together with other luminescent sensors for water quality, such as the dissolved O_2_ [50] sensor or the recently developed pH sensor [51]. In this way, a single field-deployable fiberoptic device can be used to interrogate the different sensors due to the same luminescence phase shift measurement, removing the need for different detector and optoelectronic systems [49].

## Figures and Tables

**Table 1:** Lippert–Mataga and normalized Reichardt *E*_T_^N^ parameters, absorption, and luminescence maxima, and Stokes shifts of (TBA)_4_[Ru(bpds)_3_] in selected solvents, and the corresponding emission lifetimes in air and argon-sparged solutions at room temperature.

Solvent	Δ*f*	*E*_T_^N^	λ_max,abs_ ^a^ (nm)	λ_max,em_ ^a^ (nm)	ν_abs_-ν_em_ ^b^ (cm^−1^)	τ_air_ ^c^ (ns)	τ_argon_ ^c^ (ns)
Trichloromethane	0.1504	0.259	461	598	4969	1302	2078
Dichloromethane	0.2171	0.309	462	605	5116	1386	2037
Butan-1-ol	0.2635	0.586	463	624	5573	688	1069
Acetone	0.2848	0.355	462	624	5619	666	1279
Ethanol	0.2897	0.654	461	626	5718	596	1096
Acetonitrile	0.3049	0.460	461	630	5819	434	1298
Methanol	0.3086	0.762	461	634	5919	468	822
Water	0.3199	1.000	463	635	5850	458	527

^a^ Uncertainty: ±1 nm. ^b^ Uncertainty: ±20 cm^−1^. ^c^ Uncertainty: ±0.5%.

## Data Availability

Dataset available on request from the authors.

## Supplementary Materials

The following supporting information can be downloaded at: https://www.mdpi.com/article/10.3390/s25010121/s1. Figure S1: Effect of ionic strength on the swelling of QAE Sephadex beads. Bed volumes are obtained from 1 g of dry resin as a function of the ionic strength in pH 7.6 Tris-HCl buffer with varying NaCl concentration [37]. Figure S2: (a) Relative absorption and luminescence spectra of [Ru(bpds)_3_]^4−^ in different KCl aqueous solutions. (b) Zoom on the 464 nm absorption band (left) and on the emission spectrum maxima (right) in the 0 and 500 mmol L^−1^ KCl solutions. Figure S3: Relative absorption and luminescence spectra of (TBA)_4_[Ru(bpds)_3_] in different solvents. Figure S4: Luminescence micrograph of QAE Sephadex with electrostatically immobilized Na4[Ru(bpds)_3_] into KCl solutions of 0.2 mS cm^−1^ (left) and 12.8 mS cm^−1^ (right) conductivity. Figure S5: Relative absorption and b) relative luminescence spectra of the dyed QAE Sephadex resin in different conductivity solutions (KCl). Figure S6: Stern-Volmer plot of the free [Ru(bpds)_3_]^4−^ in solution and immobilized on QAE Sephadex at 0.25 mol% in 0.2 mS cm^−1^ and 12.8 mS cm^−1^ conductivity solutions (KCl). The red lines represent the linear least squares regression. The bimolecular quenching constants calculated thereof with the Stern-Volmer equation (τ_0_/τ = 1 + *k*_q_τ_0_[O_2_]) are *k*_q_(solution) = 1.9 × 10^9^ L mol^−1^ s^−1^, *k*_q_(QAE/0.2 mS cm^−1^) = 7.2 × 10^8^ L mol^−1^ s^−1^ and *k*_q_(QAE/12.8 mS cm^−1^) = 9.0 × 10^8^ L mol^−1^ s^−1^. Figure S7: (a) Schematic representation of the conductivity sensor setup in a flow-through cell. (b) schematic representation of the sensor head. Figure S8: Sensor response function at 0.2 mS cm^−1^ (top) and 12.8 mS cm^−1^ (bottom) conductivity solutions (KCl) for different water flow rates. Figure S9: Response curves of the conductivity sensor to (a) 0.2 mS cm^−1^, (b) 0.8 mS cm^−1^, (c) 5 mS cm^−1^, (d) 9.5 mS cm^−1^, (e) 12.8 mS cm^−1^ and (f) 13.9 mS cm^−1^ solutions containing 0%, 6%, 15%, 21%, 60% and 100% O_2_ in N_2_ at different temperatures. Figure S10: ^1^H NMR spectrum of Na_4_[Ru(bpds)_3_] in DMSO-d_6_. Figure S11: MS-ESI(−) scan of Na_4_[Ru(bpds)_3_] in DMSO-d_6_. Figure S12: 1H NMR spectrum of (TBA)_4_[Ru(bpds)_3_] in acetone-d_6_. The integral of the aliphatic protons is overestimated due to co-extracting of some TBA-Cl with the Ru(II) complex. Table S1: Summary of the performance of various optical salinity or ionic strength sensors reported in the literature.
