# Supplementary material for: Luminescence Lifetime-Based Water Conductivity Sensing Using a Cationic Dextran-Supported Ru(II) Polypyridyl Complex
Source: Sensors (Basel). 2024 Dec 28;25(1):121. doi: 10.3390/s25010121 (PMC11722963; doi:10.3390/s25010121)
Supplement: Supplementary file 1 [file sensors-25-00121-s001.zip › sensors-3393789-supplementary.pdf]

Supporting Information

Luminescence Lifetime-Based Water Conductivity Sensing Using a Cationic Dextran-Supported Ru(II) Polypyridyl Complex

Ya Jie Knöbl, Lauren M. Johnston, José Quílez-Alburquerque, and Guillermo Orellana*

Chemical Optosensors & Applied Photochemistry Group (GSOLFA), Department of Organic Chemistry, Faculty of Chemistry, Complutense University of Madrid, 28040 Madrid, Spain

Chemicals and Materials

The organic solvents used (analytical or HPLC grade) were supplied by Merck (Darmstadt, Germany), Acros Organics (Geel, Belgium) or VWR (Radnor, PA, US). Reagents for synthesis were purchased from TCI (Tokyo, Japan), VWR, Merck, Fluka (Büchs, Switzerland), Alfa Aesar (Karlsruhe, Germany) or Acros. KCl (≥99%) and KNO_3 (≥98%) from VWR, NaCl (≥99.5%) and Na_2SO_4 (≥99%) from Fisher Scientific (Waltham, MA, US), $MgCl_2 \cdot 6 H_2O$ (≥99%) from Merck, $CaCl_2$ (≥95%) from Scharlab (Barcelona, Spain), and $NaNO_3$ (≥97%) from Quimipur (Madrid, Spain) were used as received. NMR solvents were from Merck, Acros or VWR and were of >98.6% isotopic purity. Type I water was obtained from a Millipore Direct-Q® 3UV purification system. N_2, argon, and O_2 of +99.995% purity from cylinders (Carburos Metálicos, Madrid, Spain) were used. N_2/O_2 mixtures were prepared with a PID Eng&Tech (Alcobendas, Spain) mass flow-controlled mixing unit.

Spectroscopic and Photophysical Measurements Equipment

Microwave-assisted synthesis was carried out on a Monowave 200 (Anton Paar, Graz, Austria). ^{1}H-NMR spectra were recorded on a Bruker AVIII HD 300 MHz BACS-60 instrument (UCM NMR Central Instrumentation Facilities). ESI spectra were recorded on a Bruker HCT Ultra ion trap mass spectrometer coupled to HPLC with ESI interface (UCM Mass Spectrometry Central Instrumentation Facilities).

All conductivity solutions were prepared using KCl unless otherwise stated. The conductivity of the solutions was measured with an Accumet AET 30 conductivity tester (Fisher Scientific).

Spectroscopic measurements were performed at (298 ± 2) K in KCl solutions contained in Suprasil® cells with a 1 cm pathlength (Hellma, Müllheim, Germany). UV-VIS absorption spectra were recorded with a Varian Cary 3Bio spectrophotometer (Palo Alto, CA, US). Corrected luminescence spectra were measured with a Horiba Fluoromax-4 spectrofluorometer (Kyoto, Japan).

Emission lifetimes (τ) were determined by single photon timing (SPT) using an Edinburgh Instruments FL-980 TCSPC spectrometer (Glasgow, UK) equipped with a Horiba NanoLed-470LH blue laser diode (463 nm, 100 kHz sub-ns pulses), a double monochromator and a red-sensitive Hamamatsu R928P photomultiplier tube detector (Hamamatsu, Japan), thermoelectrically cooled at −21 °C. The biexponential global analysis (Edinburgh Instruments FAST v. 3.5.0 analysis software) was performed by fixing the two contributing lifetimes and letting only their relative contribution change. Luminescence phase shifts were measured with a dedicated optoelectronic device Optosen® [49], which is used to simultaneously interrogate, via bifurcated fiberoptic cables (Rockwell Automation Allen-Bradley 43GR-FBS25SL, Diegem, Belgium), four sensitive terminals. Each of the 60 cm fiberoptic cable is made of a bifurcated randomized waveguide bundle (3.2 mm dia.) into a stainless steel ferrule-terminated tip. The excitation source is a high-intensity 470 nm LED digitally modulated at 156 kHz, the emission of which is passed through a 470 nm wide band-pass interference filter. The emission from the sensitive membrane at the fiber common end is monitored through an OG-590 long-pass glass filter with a red-sensitive Hamamatsu miniature photosensor module. Each measurement is averaged over a 20 point boxcar. Under standard conditions, the Optosen® device provides a resolution of 0.02°.

Confocal fluorescence microscopy images were collected with a Lumenera (Ottawa, Canada) Infinity3-1UC thermoelectrically-cooled color CCD camera (1392 x 1040 pixels) using an Olympus BX-51 microscope (Tokyo, Japan) fitted with a 10X objective. The excitation source used by the microscope is a high-intensity halogen bulb, filtered through a 463 nm band-pass interference filter. The emission from the sample is acquired through a 500 nm long-pass filter.

The four sensitive terminals were placed into a homemade flow-through PTFE cell with an internal volume of 17 μL each. Salt solutions were pumped through the flow cell using a peristaltic pump (Minipulse 2, Gilson, Madison, WI, US); the different salt solutions going through the cell were selected automatically by means of five 3-way isolation valves (NResearch, West Caldwell, NJ, US) controlled by the Optosen® unit running its programmable proprietary software. Temperature was monitored with a type K thermocouple (USB-TC01) from National Instruments (Austin, TX, US).

Table S1. Summary of the performance of various optical salinity or ionic strength sensors reported in the literature.

Optical principle	Range	Sensitivity	Remarks	Ref.
Reflectance	0 – 4 mol L^{-1}	0.0141 pm/10 mmol L^{-1}	Fiber Bragg grating with poly(imide) coating	[10]
Reflectance	0.005 – 5 mol L^{-1}	1 nm/13 mmol L^{-1} [a]	Photonic crystals	[11]
Reflectance	0.0001 – 0.01 mol L^{-1}	1 nm/0.03 log(mol L^{-1})	Photonic crystals	[13]
Fluorescence intensity	0.003 – 1 mol L^{-1}	14% /log(mol L^{-1}) [a]	Ratiometric; two dyes embedded in PEI[b]/dextran	[15]
Reflectance	0 – 0.1 mol L^{-1}	1 nm/2.5 mmol L^{-1} [a]	Coating of MA-PVP[c] polymer over mirrors	[17]
Luminescence phase shift	0.8 – 12.8 mS cm^{-1} (0.006 – 0.1 mol L^{-1})	0.063°/mS cm^{-1} (0.024°/3 mmol L^{-1})	Ru(II) polypyridyl complex embedded in cationic dextran	This work

[a] Estimated from the linear part of the calibration curve.

[b] PEI: poly(ethyleneimine).

[c] MA-PVP: polyvinylpyrrolidonedimethyl- aminoethyl methacrylate quaternized

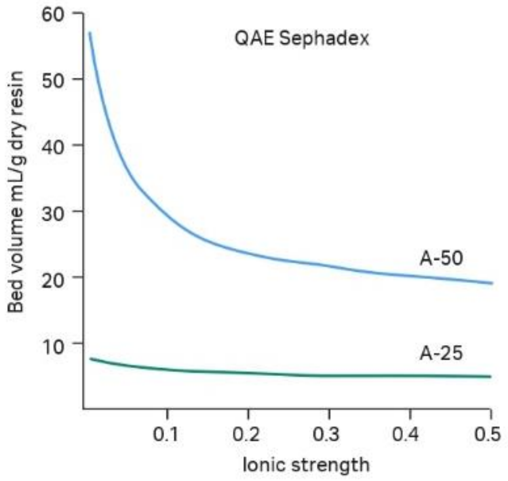

Figure S1. Effect of ionic strength on the swelling of QAE Sephadex beads. Bed volumes are obtained from 1 g of dry resin as a function of the ionic strength in pH 7.6 Tris-HCl buffer with varying NaCl concentration [37].

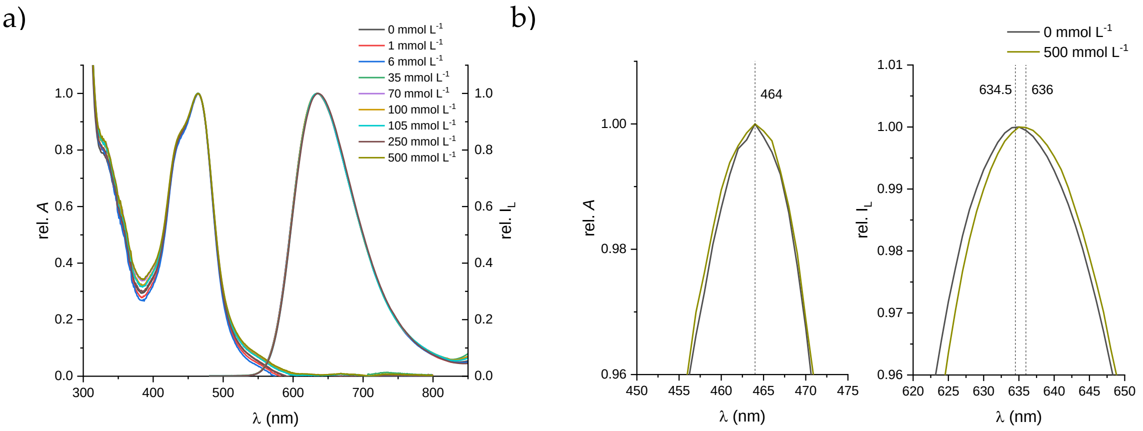

Figure S2. a) Relative absorption and luminescence spectra of $[Ru(bpds)_3]^{4-}$ in different KCl aqueous solutions. b) Zoom on the 464 nm absorption band (left) and on the emission spectrum maxima (right) in the 0 and 500 mmol L^{-1} KCl solutions.

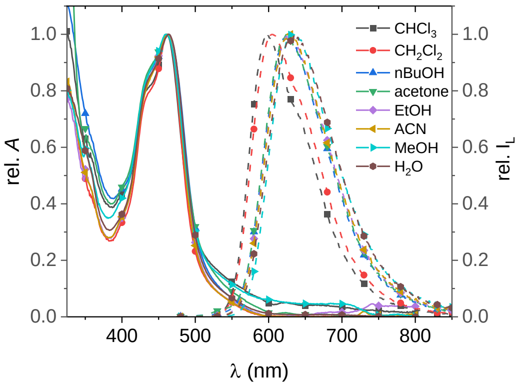

Figure S3. Relative absorption and luminescence spectra of $(TBA)_4[Ru(bpds)_3]$ in different solvents.

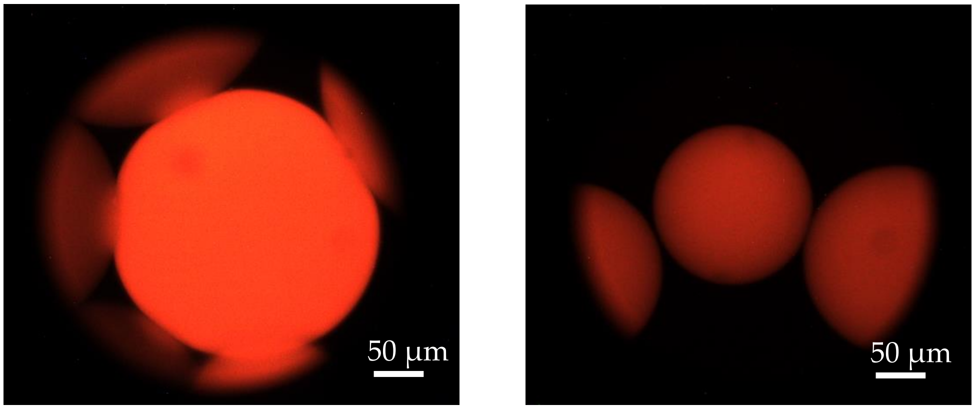

Figure S4. Luminescence micrograph of QAE Sephadex with electrostatically immobilized $Na_4[Ru(bpds)_3]$ into KCl solutions of 0.2 mS cm^{-1} (left) and 12.8 mS cm^{-1} (right) conductivity.

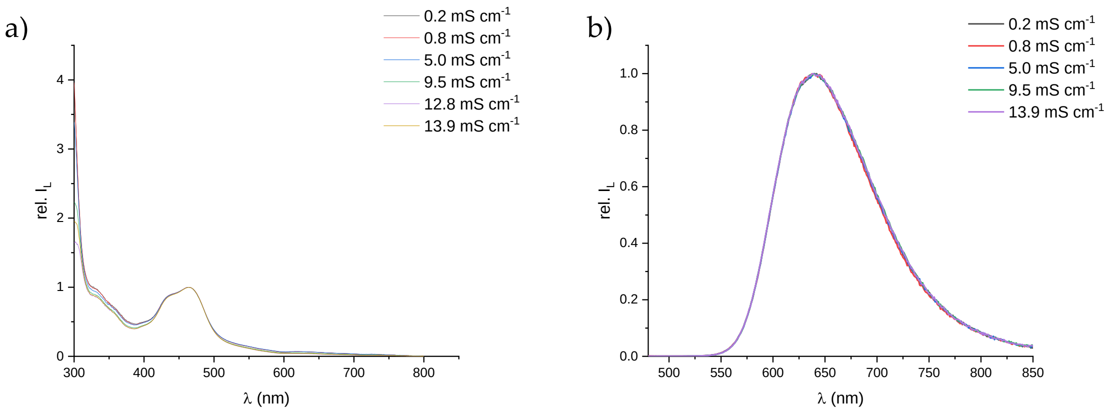

Figure S5. a) Relative absorption and b) relative luminescence spectra of the dyed QAE Sephadex resin in different conductivity solutions (KCl).

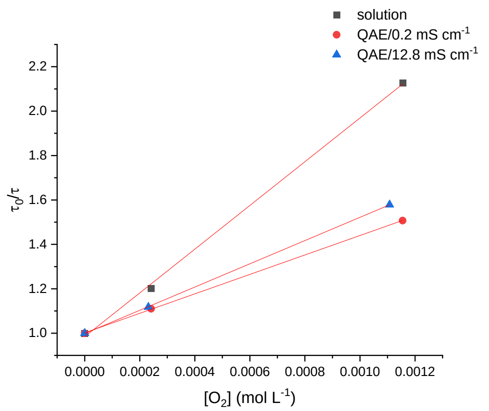

Figure S6. Stern-Volmer plot of the free $[Ru(bpds)_3]^{4-}$ in solution and immobilized on QAE Sephadex at 0.25 mol% in 0.2 mS cm^{-1} and 12.8 mS cm^{-1} conductivity solutions (KCl). The red lines represent the linear least squares regression. The bimolecular quenching constants calculated thereof with the Stern-Volmer equation ($\tau_0/\tau = 1 + k_q\tau_0[O_2]$) are k_q(solution) = 1.9 × 10^9 L mol^{-1} s^{-1}, k_q(QAE/0.2 mS cm^{-1}) = 7.2 × 10^8 L mol^{-1} s^{-1} and k_q(QAE/12.8 mS cm^{-1}) = 9.0 × 10^8 L mol^{-1} s^{-1}.

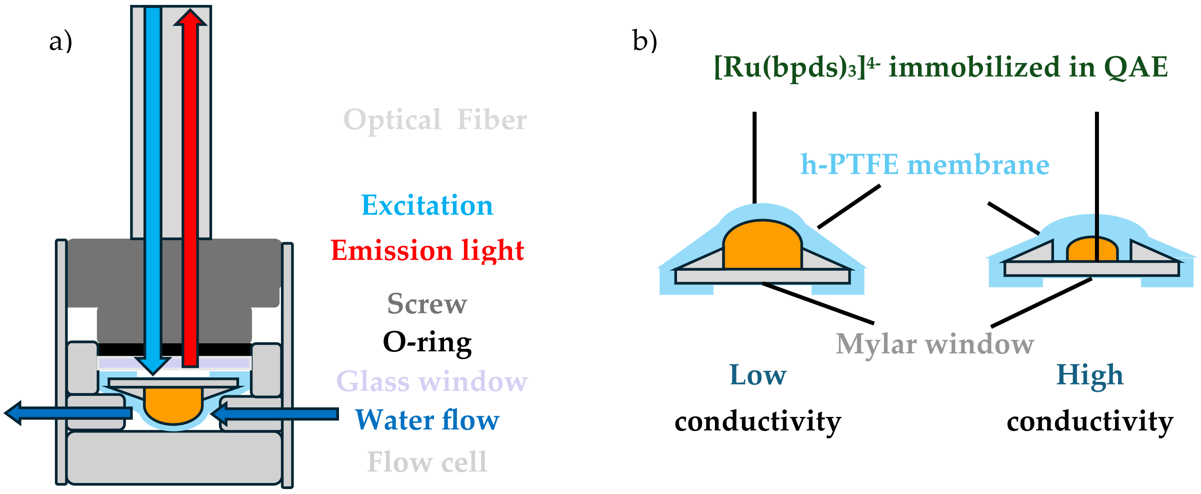

Figure S7. a) Schematic representation of the conductivity sensor setup in a flow-through cell. b) schematic representation of the sensor head.

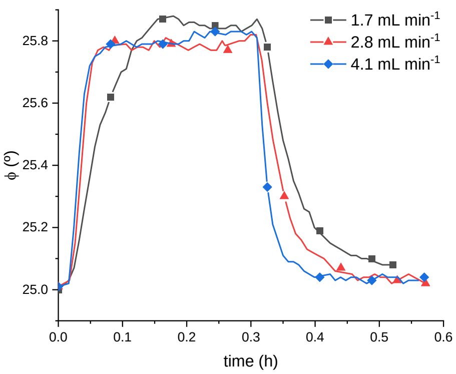

Figure S8. Sensor response function at 0.2 mS cm^{-1} (top) and 12.8 mS cm^{-1} (bottom) conductivity solutions (KCl) for different water flow rates.

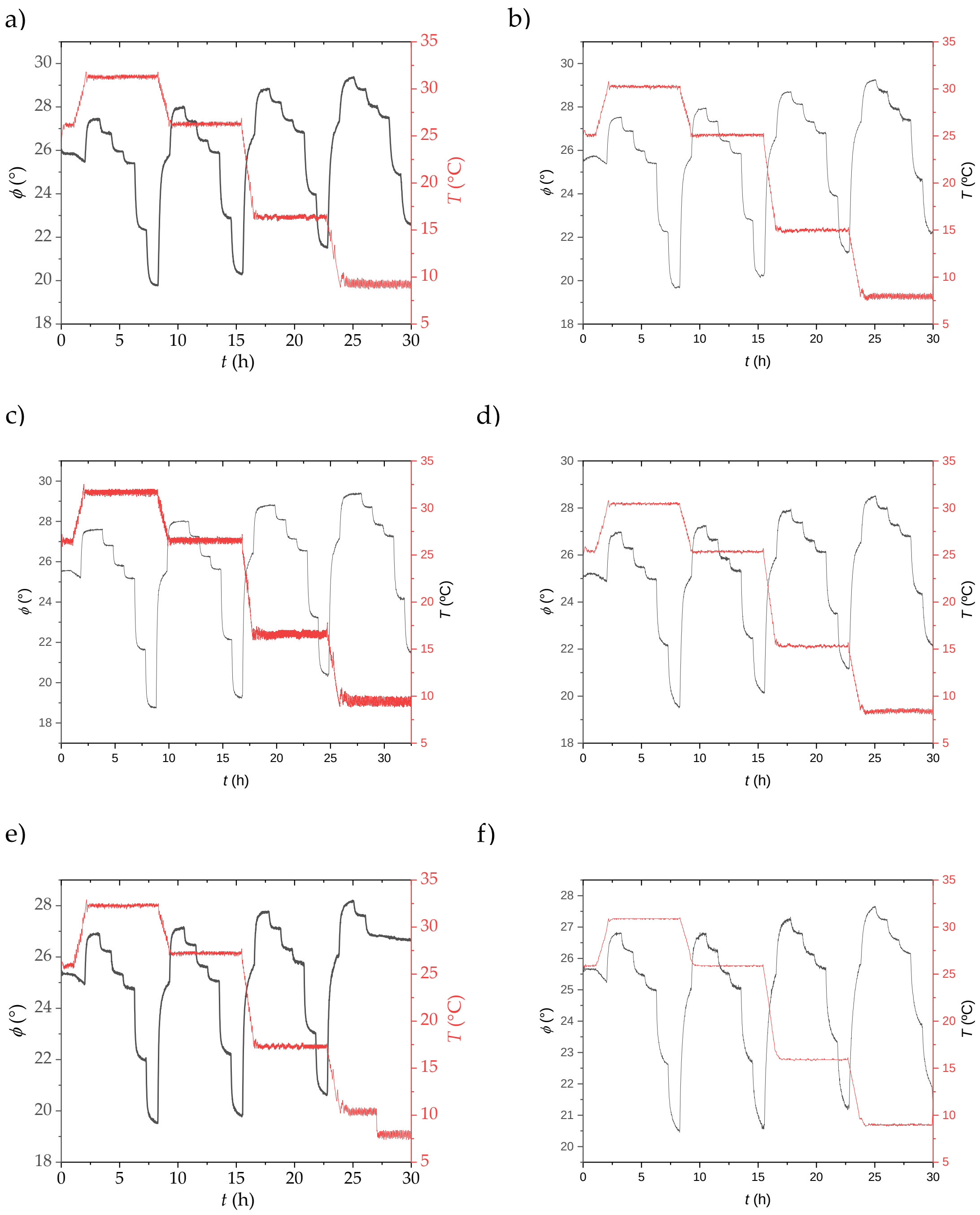

Figure S9. Response curves of the conductivity sensor to a) 0.2 mS cm⁻¹, b) 0.8 mS cm⁻¹, c) 5 mS cm⁻¹, d) 9.5 mS cm⁻¹, e) 12.8 mS cm⁻¹ and f) 13.9 mS cm⁻¹ solutions containing 0%, 6%, 15%, 21%, 60% and 100% O_2 in N_2 at different temperatures.

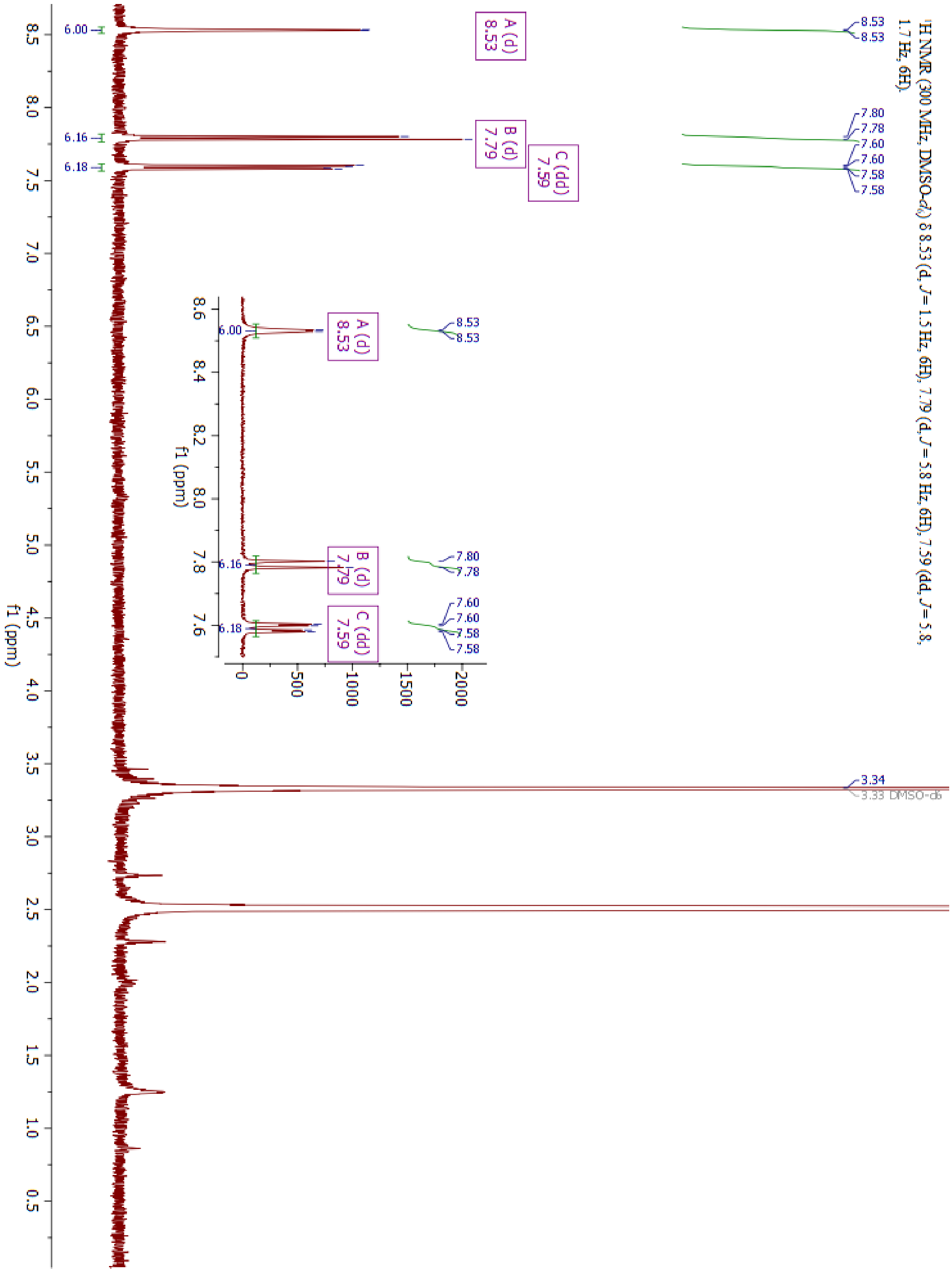

Figure S10. ^{1}H NMR spectrum of $Na_4[Ru(bpds)_3]$ in DMSO-d_6.

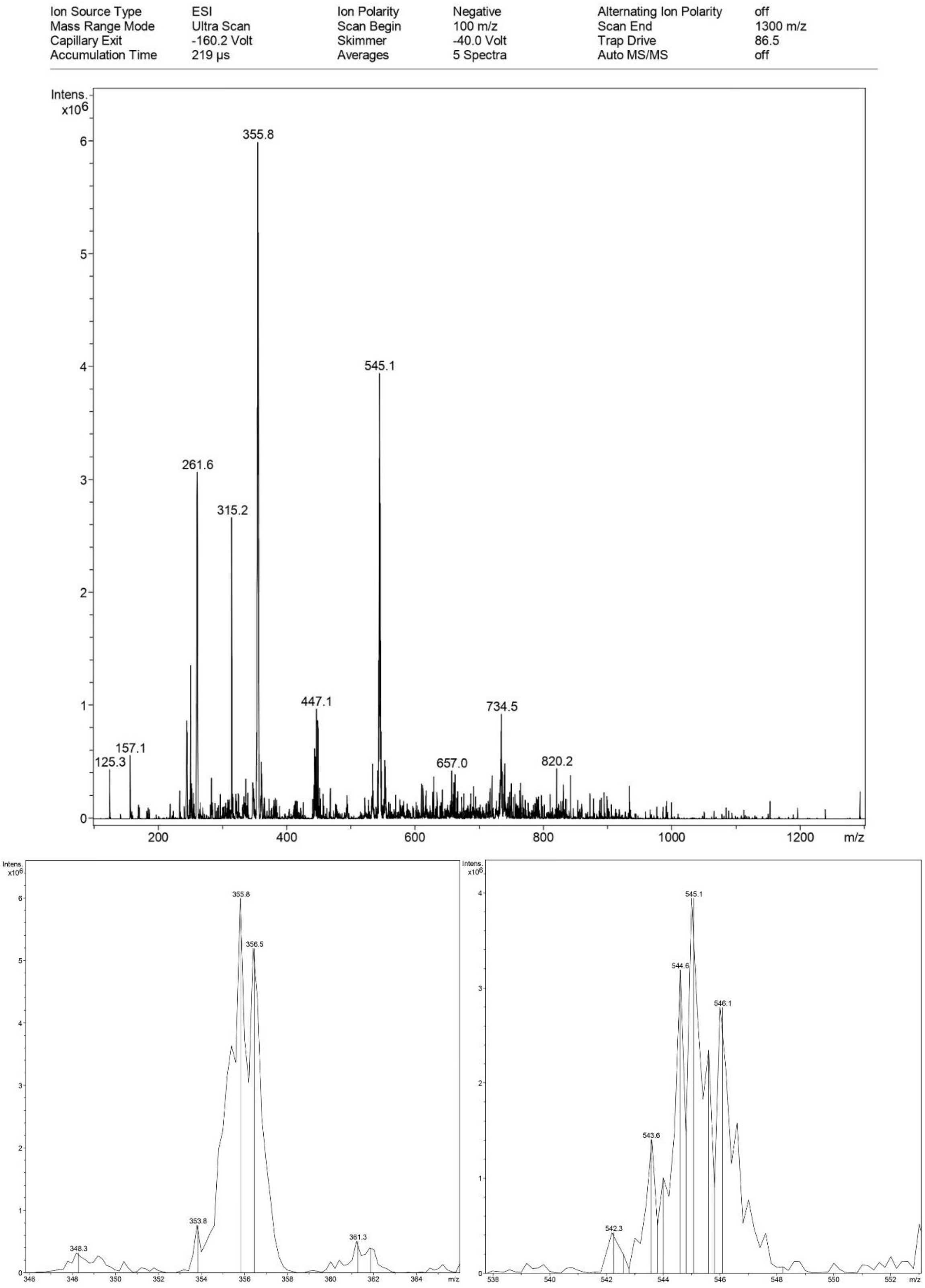

Figure S11. MS-ESI(−) scan of $Na_4[Ru(bpds)_3]$ in DMSO-d_6.

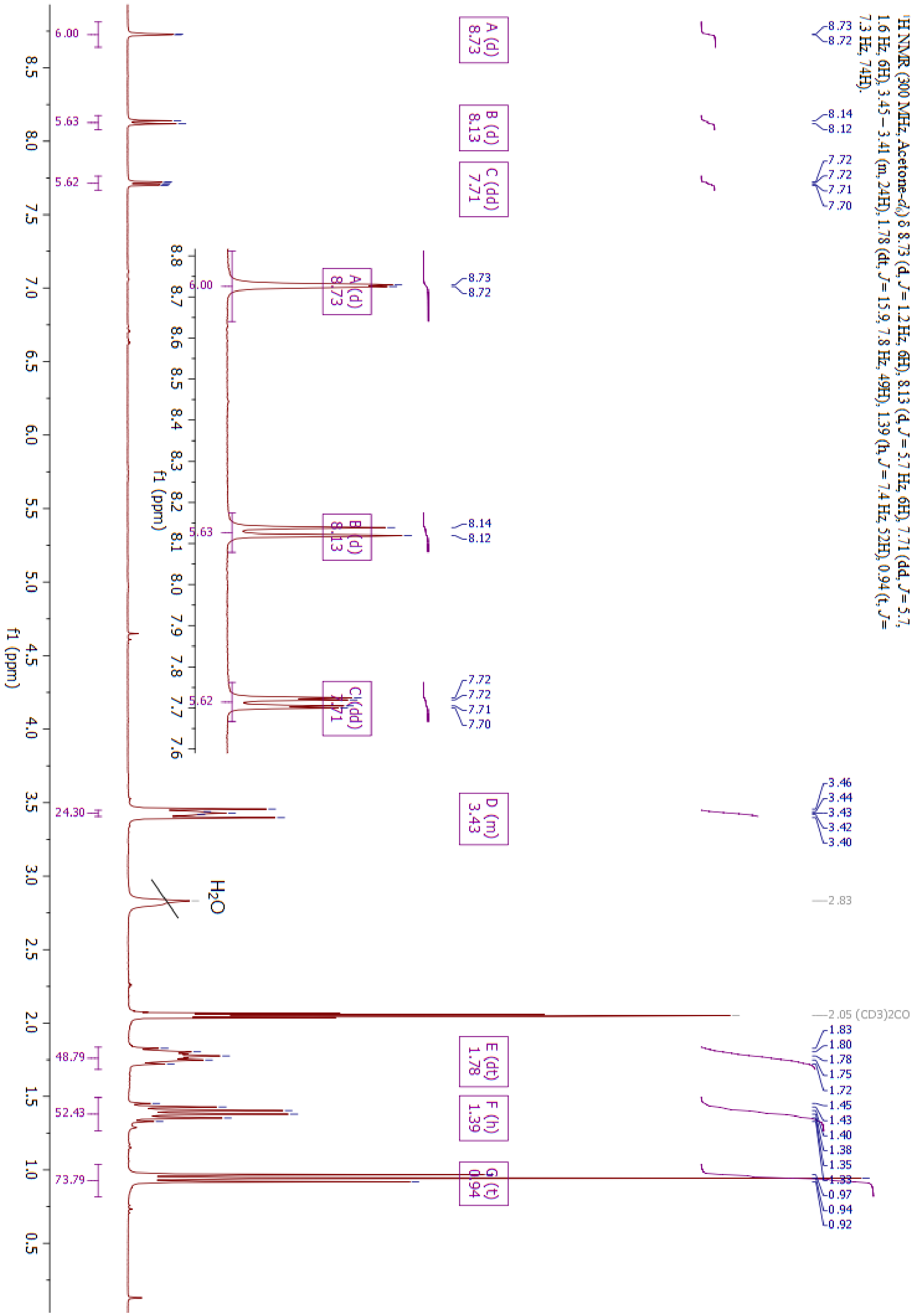

Figure S12. ^{1}H NMR spectrum of $(TBA)_4[Ru(bpds)_3]$ in acetone-d_6. The integral of the aliphatic protons is overestimated due to co-extracting of some TBA-Cl with the Ru(II) complex.

References

10. Wang, J.; Zhou, X.; Miao, Y.; Jiang, G.; Tong, L.; Tao, P.; Yu, Q.; Peng, W. Integrated and Compact Fiber-Optic Conductivity-Temperature-Depth (CTD) Sensor for Marine Detection. *Opt. Laser Technol.* **2023**, *164*, 109523, doi:10.1016/j.optlastec.2023.109523.
11. Nucara, L.; Piazza, V.; Greco, F.; Robbiano, V.; Cappello, V.; Gemmi, M.; Cacialli, F.; Mattoli, V. Ionic Strength Responsive Sulfonated Polystyrene Opals. *ACS Appl. Mater. Interfaces* **2017**, *9*, 4818–4827, doi:10.1021/acsami.6b14455.
13. Fenzl, C.; Wilhelm, S.; Hirsch, T.; Wolfbeis, O.S. Optical Sensing of the Ionic Strength Using Photonic Crystals in a Hydrogel Matrix. *ACS Appl. Mater. Interfaces* **2013**, *5*, 173–178, doi:10.1021/am302355g.
15. Christian, L.M.; Seitz, W.R. An Optical Ionic-Strength Sensor Based on Polyelectrolyte Association and Fluorescence Energy Transfer. *Talanta* **1988**, *35*, 119–122, doi:10.1016/0039-9140(88)80049-3.
17. Aussenegg, F.R.; Brunner, H.; Leitner, A.; Lobmaier, Ch.; Schalkhammer, Th.; Pittner, F. The Metal Island Coated Swelling Polymer over Mirror System (MICSPOMS): A New Principle for Measuring Ionic Strength. *Sens. Actuators B Chem.* **1995**, *29*, 204–209, doi:10.1016/0925-4005(95)01684-8.
37. Cytiva QAE Sephadex A-50 Available online: https://www.cytivalifesciences.com/en/us/shop/chromatography/resins/ion-exchange/qae-sephadex-a-50-p-02159 (accessed on 26 July 2023).
49. Urriza-Arsuaga, I.; Bedoya, M.; Orellana, G. Tailored Luminescent Sensing of NH_3 in Biomethane Productions. *Sens. Actuators B Chem.* **2019**, *292*, 210–216, doi:10.1016/j.snb.2019.04.109.
